# Exploration of the miR‐187‐3p/CNR2 pathway in modulating osteoblast differentiation and treating postmenopausal osteoporosis through mechanical stress

**DOI:** 10.1096/fj.202400113RR

**Published:** 2024-07-03

**Authors:** Lei Zhang, Canjun Zeng, Juanyu Huang, Haohang Yan, Yutao Jiang, Runguang Li

**Affiliations:** ^1^ Department of Foot and Ankle Surgery, Center for Orthopaedic Surgery, The Third Affiliated Hospital Southern Medical University Guangzhou China; ^2^ Orthopaedic Hospital of Guangdong Province Guangzhou China; ^3^ Academy of Orthopedics·Guangdong Province Guangzhou China; ^4^ Guangdong Provincial Key Laboratory of Bone and Joint Degeneration Diseases Guangzhou China; ^5^ Guangzhou Key Laboratory of Neuropathic Pain Mechanism at Spinal Cord Level Guangzhou China; ^6^ Department of Spine Surgery, Center for Orthopaedic Surgery, The Third Affiliated Hospital Southern Medical University Guangzhou China

**Keywords:** CNR2, mechanical stress, miR‐187‐3p, osteogenic differentiation, osteoporosis

## Abstract

This study aimed to explore how mechanical stress affects osteogenic differentiation via the miR‐187‐3p/CNR2 pathway. To conduct this study, 24 female C57BL/6 mice, aged 8 weeks, were used and divided into four groups. The Sham and OVX groups did not undergo treadmill exercise, while the Sham + EX and OVX + EX groups received a 8‐week treadmill exercise. Post‐training, bone marrow and fresh femur samples were collected for further analysis. Molecular biology analysis, histomorphology analysis, and micro‐CT analysis were conducted on these samples. Moreover, primary osteoblasts were cultured under osteogenic conditions and divided into GM group and CTS group. The cells in the CTS group underwent a sinusoidal stretching regimen for either 3 or 7 days. The expression of early osteoblast markers (Runx2, OPN, and ALP) was measured to assess differentiation. The study findings revealed that mechanical stress has a regulatory impact on osteoblast differentiation. The expression of miR‐187‐3p was observed to decrease, facilitating osteogenic differentiation, while the expression of CNR2 increased significantly. These observations suggest that mechanical stress, miR‐187‐3p, and CNR2 play crucial roles in regulating osteogenic differentiation. Both in vivo and in vitro experiments have confirmed that mechanical stress downregulates miR‐187‐3p and upregulates CNR2, which leads to the restoration of distal femoral bone mass and enhancement of osteoblast differentiation. Therefore, mechanical stress promotes osteoblasts, resulting in improved osteoporosis through the miR‐187‐3p/CNR2 signaling pathway. These findings have broad prospect and provide molecular biology guidance for the basic research and clinical application of exercise in the prevention and treatment of PMOP.

## Graphical abstract

The proposed mechanisms are summarized. The pathway by which miR‐187‐3p/CNR2 regulates osteoblast osteogenic differentiation for the prevention and treatment of osteoporosis is outlined. Mechanical stress, through downregulation of miR‐187‐3p, could enhance the expression of CNR2, leading to increased expression of early osteoblast markers and prevented the bone loss, which may be an alternative option to serve as a promising candidate for the prevention and treatment of PMOP in the future.

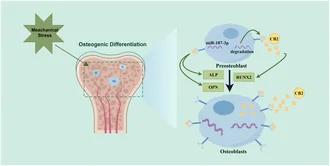

## 1 INTRODUCTION

Osteoporosis (OP) is an advancing skeletal condition characterized by decreased bone mineral density (BMD), primarily affecting postmenopausal women.^1^ Maintaining proper bone strength and integrity involves a dynamic process of continuous turnover, governed by the delicate balance between bone resorption of osteoclasts and bone formation of osteoblasts.^2^ Recently, exercise has been widely recognized as a prophylaxis for postmenopausal osteoporosis (PMOP).^3^, ^4^ During exercise, bone tissue is influenced by mechanical stresses such as tensile strain stress,^5^ compressive stress,^6^ and shear force.^7^, ^8^ This stimulation plays a crucial role in bone remodeling, promoting the differentiation of bone marrow mesenchymal stem cells (BMSCs) and osteoblast precursor cells into osteoblasts, while inhibiting the formation of mononuclear macrophages. Consequently, these cells transition into osteoclasts, leading to an increase in bone mass and ultimately maintaining bone homeostasis.^9^ This process can significantly improve the bone health of individuals with osteoporosis and enhance their overall quality of life. Osteoblasts, as pivotal effector cells in bone remodeling, have the ability to perceive mechanical stimuli both in vitro and in vivo.^10^ These stimuli can influence various intracellular signaling pathways within osteoblasts and regulate their proliferation and differentiation. Consequently, the stimulation of the stress could fine‐tuning bone remodeling to delay osteoporosis.^11^

MicroRNAs (miRNAs) are a conserved class of short, single‐stranded RNAs that play a crucial role in regulating gene expression after transcription.^12^ In our research, we conducted a treadmill experiment on rats in the early stage to further investigate the effects of exercise. The experiment confirmed that proper exercise can promote osteogenic differentiation of long osteocytes and inhibit adipose differentiation, providing an experimental basis for using exercise as a preventive and treatment measure for osteoporosis. Additionally, we performed sequencing analysis and identified miRNA with significant changes. Through PCR, we further validated miR‐187‐3p as the target miRNA.^13^ Furthermore, a notable upregulation in miR‐187‐3p expression was observed in bone samples from osteoporotic non‐fracture patients.^14^ These findings lay the groundwork for future research endeavors. Previous studies have demonstrated the involvement of miR‐187‐3p in various pathological processes. Abnormal expression of miR‐187‐3p has been observed in several tumors, including breast cancer,^15^ peripheral T‐cell lymphoma,^16^ osteosarcoma,^17^ and hepatocellular carcinoma,^18^ with implications for unfavorable clinical outcomes. Moreover, miR‐187‐3p has been implicated in the treatment of temporal lobe epilepsy,^19^ type 2 diabetes,^20^, ^21^ alleviating damage caused by ischemia–reperfusion,^22^, ^23^ and promoting the growth of human aortic smooth muscle cells for the prevention and treatment of cardiovascular diseases.^24^ In the context of bone homeostasis regulation, studies have indicated a decrease in miR‐187‐3p expression after treating bone cells with titanium.^25^ Research has revealed that miR‐187‐3p inhibits the osteogenic differentiation of human osteoblast precursor cells (hFOB 1.19) by suppressing cannabinoid receptor 2.^26^ Additionally, another study found that circ‐Iqsec1 induces BMSC osteogenic differentiation through the miR‐187‐3p/Satb2 signaling pathway.^27^ Nevertheless, the role and mechanism of miR‐187‐3p in OP have not been investigated.

Cannabinoid receptor type 2 (CNR2) is a crucial receptor of the endocannabinoid system that plays a major role in maintaining bone mass homeostasis.^28^ A previous study demonstrated that depleting both CNR1 and CNR2 in female mice inhibited age‐related bone loss, while deficiency of individual receptors could be detrimental.^29^ Furthermore, 17‐β estradiol was found to restrain osteoclast activity by upregulating the expression of the CNR2.^30^ Additionally, activation of CB2 receptor has been shown to enhance mesenchymal stem cell osteogenic differentiation^31^, ^32^ Notably, CB2 receptor has been predicted as a potential target of miR‐187‐3p, which is believed to have significant implications in miR‐187‐3p mediated osteogenic differentiation.^26^ Therefore, CB2 receptor signals hold promise for the prevention and treatment of osteoporosis.

However, the mechanism by which exercise prevents loss of bone mass remains undefined. Our results collectively demonstrate the significant role of the miR‐187‐3p/CNR2 axis in osteoblastic differentiation. In order to identify an exercise regimen that targets the miR‐187‐3p/CNR2 pathway and holds promise in the treatment of osteoporosis, we compared the training regimen in ovariectomized (OVX) C57BL/6 mice to the strain stress applied to mouse embryo osteoblast precursor cells.

## 2 MATERIALS AND METHODS

### 2.1 Animal experiment

All animal protocols were approved by the Institutional Animal Care and Use Committee (IACUC) at the Southern Medical University before the study, and all animal experiments were performed in accordance with Institutional Animal Care and Use Committee guidelines. A total of 24 8‐week‐old female C57BL/6 mice (NO. NFYY‐2019‐58) were obtained from the Experimental Animal Center of Southern Medical University in Guangzhou, China. A running exercise for the mice was performed using a laboratory animal treadmill (Zhenghua ZH‐PT, China). Bilateral ovariectomy (OVX, *n* = 12) or sham operation (Sham, *n* = 12) was carried out on the mice under anesthesia using 2% tribromoethanol (20 μL/g of body weight, Cat#T831042, Macklin, Shanghai, China) via intraperitoneal injection. The OVX surgery was conducted following previously described methods.^33^, ^34^ After 2 weeks, the mice were randomly divided into two subgroups: OVX‐sedentary (OVX) and OVX‐exercise (OVX‐EX) groups, as well as Sham‐sedentary (Sham) and Sham‐exercise (Sham‐EX) groups. To acclimate the mice to the treadmill, they underwent 5 days of exercise for 30 min per day at 6 m/min on a 25% slope, followed by 2 days of rest. Over the next 8 weeks, the running time and speed were gradually increased, reaching 60 min of exercise per day at 15 m/min on a 25% slope.^35^ After the training exercise, all the mice were euthanized, and the uterus was removed and weighed. Bilateral femur were collected for histological and micro‐CT analysis. Furthermore, the weight of the mice was measured before and after the running program.

### 2.2 Primary osteoblast culture

Newborn C57BL/6 mice were obtained from the Experimental Animal Center of Southern Medical University (Guangzhou, China). Primary osteoblasts were isolated from mice calvariae as previously described.^36^, ^37^ The digestion process involved using 0.25% trypsin (Cat#25200056, Gibco, USA) for 20 min, followed by two rounds of digestion with 0.1% type II collagenase (Cat#17101015, Gibco, USA) in the minimum essential medium (αMEM, Cat#C12571500BT, Gibco, USA) for 60 min each. The cells obtained from the third digestion were then suspended in the αMEM growth medium supplemented with 10% fetal bovine serum (FBS; Cat#04–001‐1A, Biological Industries, Israel), 100 U/mL penicillin, and 100 mg/L streptomycin (Cat#15140148, Gibco, USA). These cells were cultured in a 10 cm^2^ petri dish at 37°C in a 5% CO_2_ moist atmosphere until they reached confluence. The growth medium was changed after 24 h, and subsequently every other day. For all experiments, cells between the second and third generations were used.

### 2.3 Application of mechanical stress to cells

A total of 1 × 10^5^ cells/mL were cultured on a six‐well silica gel membrane plate coated with type I collagen (Cat#UF‐4001C, Flexcell International, USA).^38^ After 24 h of culture to reach 90% confluency, at which time the growth medium was replaced with αMEM osteogenic induction solution to continue culture for 24 hours, followed by the application of cyclic tensile stress. The mechanical strain was applied to the collagen‐coated plates using the Flexcell FX‐5000 system (Cat#FX‐5000, Flexcell International, USA) with a cyclic tensile stress of 10% of the tensile stress and a frequency of 0.5 Hz for 6 h per day.^39^ After the experimental processes were concluded, the cells were gathered for subsequent analyses. Control groups were cultured under similar conditions but without stretching. This experimental setup aimed to investigate the effects of cyclic strain stress (CTS) on osteoblasts using an osteogenic medium. The αMEM osteogenic induction solution consisted of growth medium supplemented with 50 μg/mL ascorbic acid, 10 mM β‐glycerophosphate, and 1 nM dexamethasone (DXM, Cat#AAPR99‐C500, Pythonbio, China).

### 2.4 Primary osteoblasts transfection

Primary osteoblasts were seeded on six‐well silicone rubber culture plates at a density of 1.0 × 10^5^ cells/mL. They were then incubated in αMEM containing 10% FBS at 37°C in a 5% CO_2_ incubator. Subsequently, the osteoblasts were transfected with the mmu‐miR‐187‐3p mimic (50 nM, Cat#miR10000216‐1‐5, RIBBIO, China), mmu‐miR‐187‐3p inhibitor (100 nM, Cat#miR20000216‐1‐5, RIBBIO, China), and their corresponding negative controls (Cat#miR1N0000002‐1‐5 and Cat#miR2N0000 002‐1‐5, RIBBIO, China) using Lipofectamine‐3000 (Cat#Lip3000, Invitrogen, USA). The cells were also transfected with pcDNA3. 1‐CNR2 (100 ng/μL, Cat#XM015, Tsingke Biotech, China) (GenePharma, Shanghai, China) using Lipofectamine 3000 (Invitrogen, USA) following the manufacturer's protocol. After 24 h, the cyclic tensile stress group (CTS) was exposed to CTS (10%, 0.5 Hz, 6 h/day). The growth medium groups (GM) were cultured without CTS. On the seventh day, osteoblasts were collected for real‐time quantitative PCR (RT‐qPCR) and western blot (WB) analyses.

### 2.5 Alkaline phosphatase staining

Phosphate‐buffered saline (Cat#SH0058‐1, PBS; Sihe Biology, Guangzhou, China) was used for washing the cells, followed by fixation with 4% paraformaldehyde (PFA, Cat#SH268C, Sihe Biology, Guangzhou, China). After another round of PBS washing, BCIP/NBT Alkaline Phosphatase Color Development Kit staining (Cat#C3206, Beyotime, Shanghai, China) was conducted. The cells were incubated at 37°C for 30 min and then washed twice with PBS. Subsequently, the cells were observed using an optical microscope after washing with PBS.

### 2.6 Histological assessment, immunohistochemistry, and immunofluorescence

Isolated femoral bones were fixed in 4% PFA formalin, decalcified using 10% EDTA (Cat#SH5546, Sihe Biology, Guangzhou, China), embedded in paraffin, and then cut into 5 μm sections. Subsequently, these sections were deparaffinized and stained with hematoxylin and eosin (HE, Cat#C0105S, Beyotime, China) as well as Masson (Cat#G1346‐8, Solarbio, China), enabling the histomorphometry analysis of cancellous bone and bone marrow.

Immunohistochemistry and immunofluorescence were performed as previously described.^40^ For immunohistochemistry, after antigen retrieval and blocking, the sections were probed with a primary antibody against CNR2 (1:100, Cat#bs‐2377, Bioss, China). After incubation with the corresponding secondary antibodies, color development was performed with diaminobenzidine (DAB, Cat#34002, Thermo Scientific, China) and counterstained with hematoxylin (Cat#C0105S, Beyotime, China).

For immunofluorescence, the sections were probed with a primary antibody against CNR2 (1:100, Cat#bs‐2377, Bioss, China) at 4°C overnight. The sections were then washed three times in PBS and incubated with an Alexa Fluor secondary antibody (1:500, Cat#A23620, Abbkine, China) at room temperature for 1 h. Nuclei were stained with DAPI (Cat#S2110, Solarbio, China), and specimens were imaged under an inverted Olympus FV1000 laser‐scanning confocal microscope (Olympus, Tokyo, Japan). The CNR2 staining intensity and fluorescence intensity were measured with ImageJ (Wayne Rasband, NIH, USA) software.

### 2.7 Microcomputed tomography analysis

The femoral bones were scanned using high‐resolution μCT40 (Scanco Medical, Wangen‐Bruttisellen, Switzerland). We reconstructed and analyzed images using Image Processing Language v.5.15 software (SCANCO Medical, Switzerland). The parameters, including trabecular bone volume fraction (BV/TV), trabecular number (Tb.N), and trabecular spacing (Tb.Sp) and trabecular thickness (Tb.Th).

### 2.8 Real‐time quantitative PCR analysis

Total RNA was extracted from osteoblasts and distal femur using Trizol (Cat#YBS‐15596026, Invitrogen, Carlsbad, CA), and miRNA was isolated using MirVana miRNA separation kit (Cat#AM1561, Invitrogen, Carlsbad, CA) according to the manufacturer's rapid extraction procedure. Bone tissue was ground into small particles with liquid nitrogen and added to Trizol, then transferred to a tissue grinder (Cat#JXFSTPRP‐CL, JINGXIN, Shanghai, China). Subsequently, it was converted into complementary DNA (cDNA) through reverse transcription using the Prime Script reverse RT Master Mix kit (Cat#RR037A, Takara, China). The miR‐187‐3p and U6 specific RT stem‐loop primer were purchased from Ribobio (Cat#ssD809230195 and ssD0904071008, RiboBio, China). The obtained cDNAs were used as templates for PCR amplification to detect expression levels of miR‐187‐3p and target genes.

The mRNA levels were detected using TB Green® Premix Ex Taq™ II (Cat#RR820A, Takara, China) and the LightCycler® 96 PCR System (Roche, Switzerland) through RT‐qPCR. All primer sequences (5′‐3′) were shown as following: miR‐187‐3p forward TCGTGTCTTGTGTTGCAGCC, reverse GTGCAG GGTCCGAGGT; Alkaline phosphata (ALP) forward CGTCTCCATGGTGGATTATGCT, reverse TGGAT GTCCTTTACCGTCGTG; Runt‐related transcription factor 2 (Runx2) forward TGGCTTGGGTTTCAGGTTAG, reverse GGTTTCTTAGGGTCTTGGAGTG; Oseteopontin (OPN) forward TCCCTCGATGTCATCCCTGT, reverse ATCACATCCGACTGATCGGC; CNR2 forward AGCCCTCATACCTGTTCATTGG, reverse GTGAAGGTCATAGTCACGCTG; GADPH forward AGGTCGG TGTGAACGGATTTG, reverse TGTAGACCATGTAGTTGAGGTCA; U6 forward CGCTTCGGCAGCACATATACTAAAATTGGAAC, reverse GCTTCACGAATTTG CGTGTCATCCTTGC. The 2^−∆∆Ct^ method was used for the quantification of relative mRNA levels. The U6 and GADPH level was considered as a reference.

### 2.9 Western blot

The cell proteins were extracted in RIPA lysis buffer (Cat#P0013B, Beyotime, China) with PMSF (Cat#FD0100, Fdbio science, China). However, the bone tissue was initially ground into small particles with liquid nitrogen before being added to the RIPA buffer. Subsequently, magnetic beads were used to further grind the tissue in a tissue grinder (Cat#JXFSTPRP‐CL, JINGXIN, Shanghai, China). Following quantification, the proteins were separated by SDS‐PAGE electrophoresis and transferred onto PVDF membranes (0.22 μm, Cat#ISEQ00010, Merck Millipore, Germany). Then, membranes were incubated with primary antibodies and horseradish peroxidase (HRP) conjugated secondary antibody. Protein signals were detected by the ECL solution (Cat#FD8001, Fdbio science, China). Following the manufacturer's instructions, immunolabeled proteins were detected using the GeneGnome XRQ detection system (Syngene, UK) as instructed. The protein band strength was quantified using ImageJ software (Wayne Rasband, NIH, USA) normalizing against GAPDH. The used primary antibodies were listed below: CNR2 antibody (Cat#DF8646, Affinity China), OPN antibody (Cat#AF0227, Affinity, China), ALP antibody (Cat#DF6225, Abclonal, China), and Runx2 antibody (Cat#AF5186, Affinity, China).

### 2.10 Statistical analysis

All data were presented as means ± standard deviation (*SD*). The statistical analysis was undertaken using GraphPad Prism software (version 8.0), and the significant difference was determined with one‐way ANOVA analysis of variance or two independent samples *t*‐test. Differences were considered significant when *p* below .05.

## 3 RESULTS

### 3.1 OVX mice combined with treadmill exercise can delay osteoporosis

The uterine weight in the OVX group was significantly lower than that in the Sham group, indicating successful ovariectomy in the OVX group (Figure 1A–D). Previous research has indicated that higher weight could potentially act as a protective factor against osteoporosis. To mitigate the impact of weight on the treadmill model of mice, 10 week‐old mice were weighed prior to the treadmill test. The findings revealed no notable variance in body weight across the four groups, effectively removing the influence of body weight on the mouse treadmill model. (Figure 1E).

**FIGURE 1:**
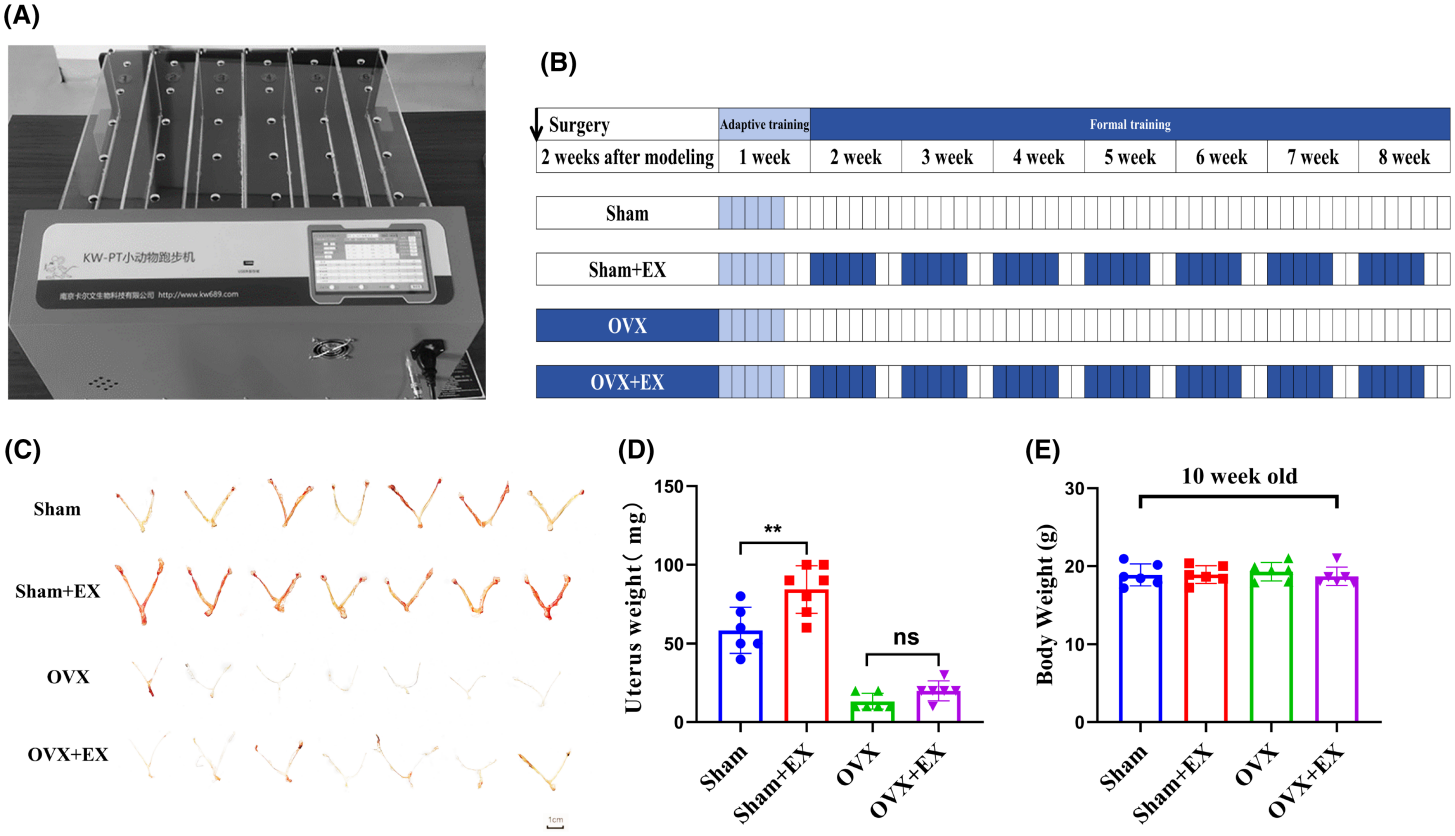
Study design. (A) This figure shows a picture of the treadmill apparatus used in the study. (B) The schematic provides an overview of the experimental protocol. Female C57 mice were randomly divided into four groups. At 8 weeks of age, mice underwent either OVX or Sham surgery and were given 2 weeks to recover. At 10 weeks of age, the mice were subjected to treadmill exercise. (C) After 8 weeks of running or being allowed to move freely in their cages, the uteruses of the mice were harvested. (D) The weights of the uteruses were significantly decreased after OVX, regardless of running. The Sham group (*n* = 12) and the OVX group (*n* = 12). (E) This section lists the body weight of mice 2 weeks after modeling. The values shown are the average of SEM. There was no significant change in body weight at 2 weeks after ovariectomy (called “10 week old”, Purge 0.086).**p < .01; ns, no significant difference.

### 3.2 Effects of treadmill exercise on trabeculae and bone mass in OVX mice

To evaluate the impact of treadmill exercise on preventing osteoporosis, we utilized micro‐CT to analyze the bone mass and structure of the femur in each group. Our findings revealed that the bone mass in the two trabeculae of osteoporotic mice was lower compared to Sham‐operated mice. This decrease was attributed to a significant reduction in BV/TV, Tb.N, Tb.Th, and Tb.Sp, along with a notable increase in Tb.Sp in osteoporotic mice. However, after 9 weeks of treadmill running, there was a significant increase in BV/TV, Tb.N, Tb.Th, and Tb.Sp, while Tb. Sp decreased in both the Sham and OVX groups (Figure 2A–E). Additionally, HE and Masson staining demonstrated improved trabeculae after treadmill running in the Sham and OVX groups (Figure 2F,G). These findings suggest that during the progression of osteoporosis, the decrease in bone mass coincides with a decline in bone formation ability. Treadmill exercise promotes bone formation ability and increases bone mass in aging mice, thereby aiding in the prevention of osteoporosis.

**FIGURE 2:**
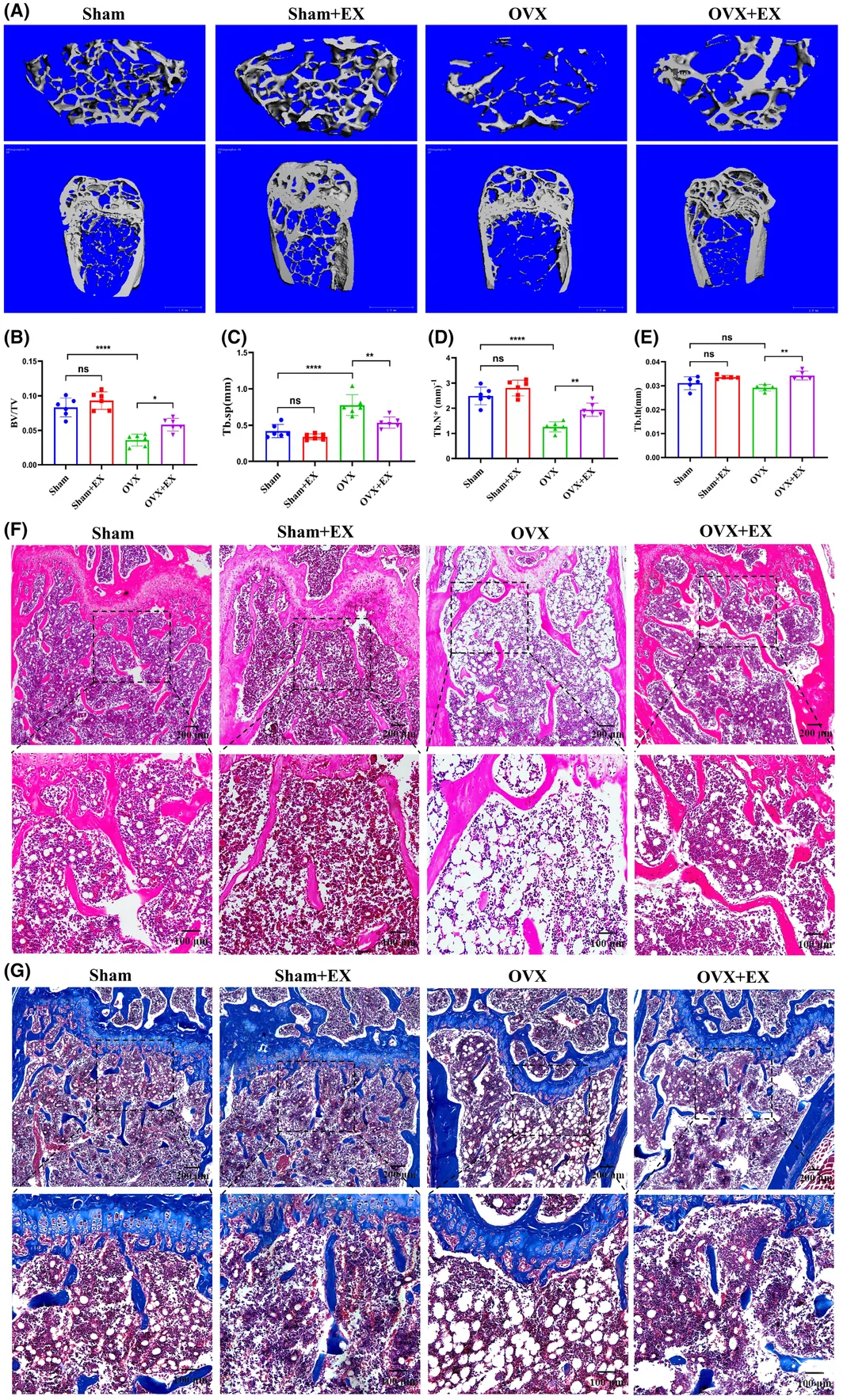
Effects of treadmill exercise on trabeculae and bone mass in OVX mice. (A) Representative 2D and 3D micro‐CT images of mice femur. (B–E) Quantitative analysis of bone structural parameters: trabecular bone volume fraction (BV/TV), trabecular number (Tb.N), trabecular thickness (Tb.Th), trabecular separation (Tb.Sp). (F–G) Representative images of H&E and Masson stained decalcified femoral sections. Scale bars: 200 and 100 μm. **p < .01 and ****p < .0001; ns, no significant difference.

### 3.3 Effect of treadmill exercise on the expression of miR‐187‐3p and CNR2 in bone of mice

Following ovariectomy, the expression of miR‐187‐3p significantly increased in the femur of ovariectomized mice, while the expression of CNR2 mRNA significantly decreased. However, after 8 weeks of treadmill exercise, both the OVX and Sham groups showed a significant decrease in the expression of miR‐187‐3p, while the expression of CNR2 mRNA significantly increased in OVX mice (Figure 3A). Moreover, the expression of CNR2 protein in the femur of ovariectomized mice was significantly lower than that of Sham mice. However, 8 weeks of treadmill exercise led to a significant increase in the expression of CNR2 protein in both the OVX and Sham mice (Figure 3B). To assess the expression of CNR2 in femur tissue, we performed immunohistochemistry as we hypothesized that CB2 receptor may play a crucial role in promoting osteogenic differentiation. The expression of CB2 receptor in the trabecula of the distal femur was lower in the OVX group compared to the Sham group, but the expression of CNR2 protein was enhanced in the Sham + EX and OVX + EX groups (Figure 3C). Additionally, we conducted immunohistochemistry and immunofluorescence staining for CNR2 on the femurs of mice to examine the correlation between CNR2 and osteoporosis. The results revealed that the bone marrow of the OVX group displayed weaker CNR2 staining and fluorescence intensity compared to the Sham group, which was reversed by treadmill exercise (Figure 3D,E).

**FIGURE 3:**
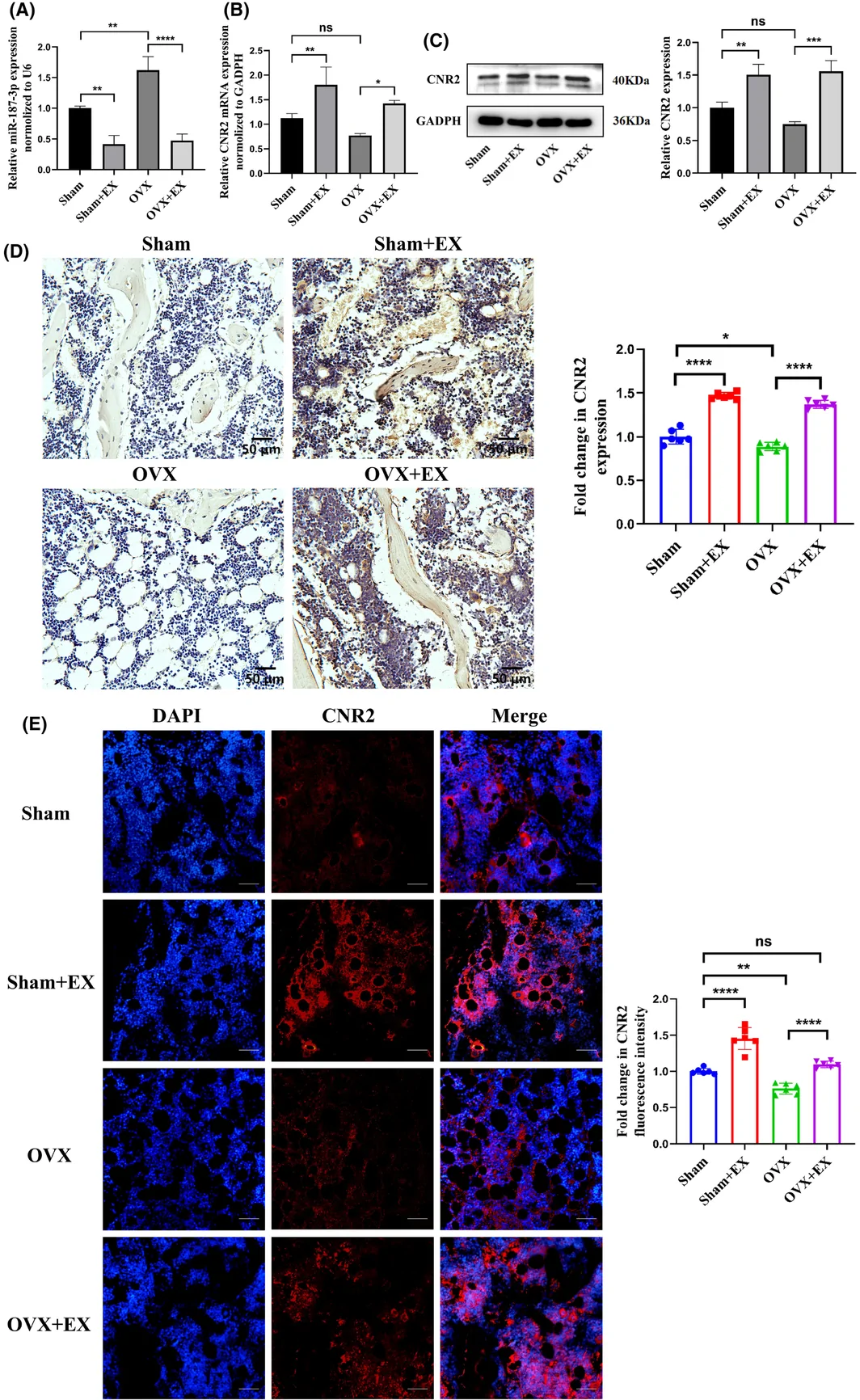
Effects of treadmill exercise on the expression of miR‐187‐3p and CNR2 in bone tissue of OVX mice. The mice underwent treadmill training for 8 weeks. (A–C) The protein and mRNA levels of CNR2 and miR‐187‐3p were determined using western blotting and real‐time RT‐qPCR, respectively. The expression of miR‐187‐3p and CNR2 mRNA, and the level of CNR2 protein were measured. (D) Immunohistochemical staining and CNR2 staining were used to observe changes in tissue structure and protein expression. (E) Immunofluorescence staining of CNR2 revealed that treadmill exercise increased the expression level of CNR2. Scale bars: 50 μm. *p < .05, **p < .01, and ****p < .0001; ns, no significant difference.

### 3.4 Osteogenic differentiation induced by CTS depends on expression and activation of miR‐187‐3p/CNR2 pathway

This study explored the association between cyclic tensile stress and osteogenic differentiation of osteoblasts. First, osteoblasts were exposed to cyclic tensile stress for 0, 3, and 7 days. Protein and RNA expression of Runx2, OPN, and ALP, as markers of osteogenic differentiation, increased after 7 days under cyclic tensile stress (Figure 4A–D). Similar results were obtained by ALP staining of osteoblasts, which was higher under cyclic tensile stress (Figure 4E). These results suggest that cyclic tensile stress promotes cell early stage of osteogenic differentiation in osteoblasts.

**FIGURE 4:**
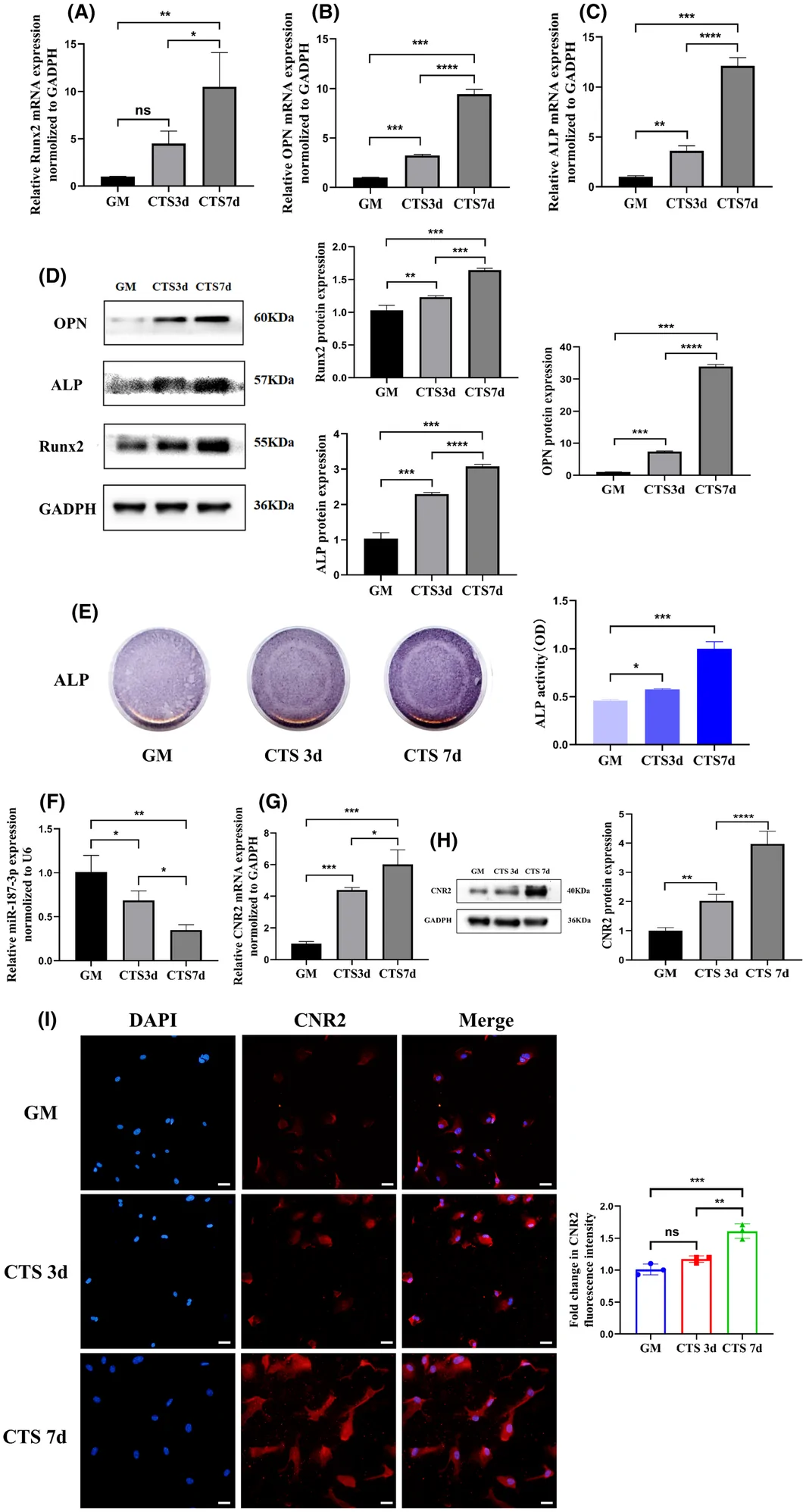
Osteogenic differentiation induced by CTS depends on expression and activation of miR‐187‐3p/CNR2 pathway. (A–C) The mRNA expression of Runx2, OPN, and ALP was measured after 3 and 7 days of CTS. (D) The protein expression of Runx2, OPN, and ALP was also measured after 3 and 7 days of CTS. (E) The ALP staining and quantitative analysis was performed after 3 and 7 days of CTS. (F‐G) The mRNA expression of miR‐187‐3p and CNR2 was measured after 3 and 7 days of CTS. (H) The protein expression of CNR2 was also measured after 3 and 7 days of CTS. (I) Immunofluorescence of CNR2 was performed after 7 days of CTS, with a scale bar of 50 μm.*p < .05, **p < .01, ***p< .001 and ***p < .0001; ns, no significant difference.

We also explored whether cyclic tensile stress regulates expression of osteogenic differentiation via the miR‐187‐3p/CNR2 pathway. We utilized RT‐qPCR, western blot, and immunofluorescence techniques to analyze the expression of miR‐187‐3p and CNR2 in osteoblasts. RT‐qPCR showed that the expression of miR‐187‐3p was inhibited under cyclic tensile stress (Figure 4F). Conversely, western blotting and RT‐qPCR showed that expression of CNR2 increased in the CTS group (Figure 4G,H). The results of immunofluorescence showed that expression of CNR2 significantly increased under cyclic tensile stress (Figure 4I). These results showed that cyclic tensile stress upregulated CNR2 via downregulating miR‐187‐3p expression. The above experiments show that cyclic tensile stress regulates the early stage of osteogenic differentiation via the miR‐187‐3p/CNR2 pathway.

### 3.5 Cyclic tensile strain and overexpression of CNR2 reversed the inhibitory effect of miR‐187‐3p on osteogenic differentiation of osteoblasts

The experiments above have demonstrated that miR‐187‐3p was downregulated in osteoblasts undergoing osteogenic differentiation. The functional influence of miR‐187‐3p in osteogenic differentiation of osteoblasts was further assessed. As shown in (Figure 5A–D), the expression of Runx2, OPN, and CNR2 was significantly restrained in miR‐187‐3p‐overexpressing osteoblasts following culture at 37°C for 48 h. Western blot assay showed that expression levels of Runx2, OPN, and CNR2 were decreased in miR‐1873p‐overexpressing osteoblasts after culture at 37°C for 48 h. Conversely, their levels were upregulated in miR‐187‐3p‐silenced osteoblasts undergoing osteogenic differentiation (Figure 5E). Moreover, ALP staining and ALP activity detection indicated that upregulation or downregulation of miR‐187‐3p inhibited or accelerated osteogenic differentiation, respectively, in osteoblasts undergoing osteogenic differentiation (Figure 5F).

**FIGURE 5:**
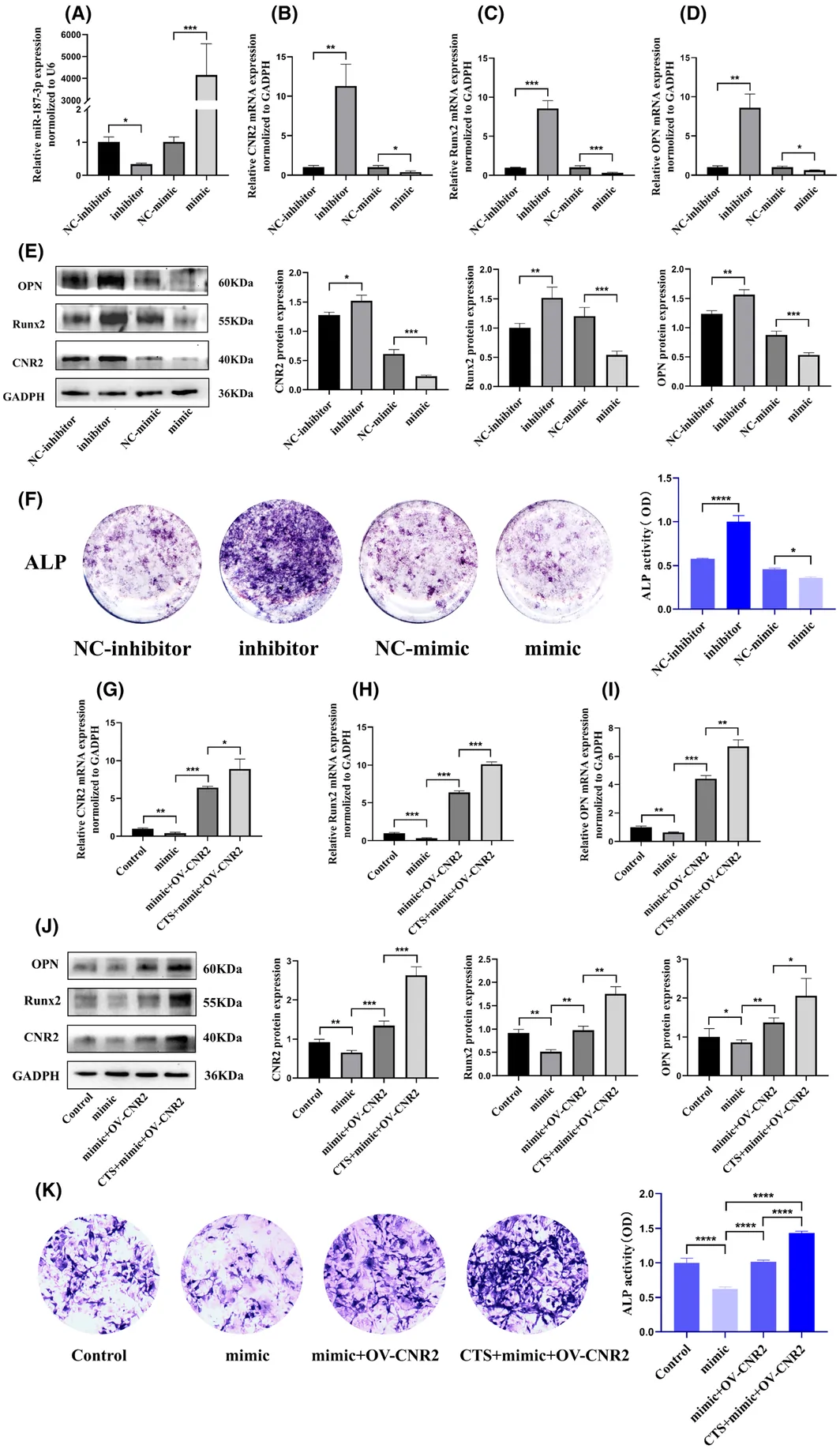
Cyclic tensile strain and overexpression of CNR2 reversed the inhibitory effect of miR‐187‐3p on osteogenic differentiation of osteoblasts. (A–D) The expression levels of miR‐187‐3p, CNR2, Runx2, and OPN were measured in osteoblasts undergoing osteogenic differentiation with upregulation or downregulation of miR‐187‐3p. (E) Western blot assay was performed to analyze the expression levels of OPN, Runx2, and CNR2 in osteoblasts undergoing osteogenic differentiation with silencing or knockdown of miR‐187‐3p. (F) ALP staining and quantitative analysis detection in osteogenic differentiated osteoblasts following miR‐187‐3p upregulation or downregulation. (G–I) The expression levels of Runx2, OPN, and CNR2 were analyzed in osteogenic differentiated osteoblasts following CNR2 upregulation and/or miR‐187‐3p upregulation with/without CTS. (J) The expression levels of Runx2, OPN, and CNR2 in osteogenic differentiated osteoblasts were analyzed using western blot assay. (K) ALP staining and quantitative analysis detection in osteogenic differentiated osteoblasts following CNR2 upregulation and/or miR‐187‐3p upregulation with/without CTS. Results were presented as mean ± *SD*. *p < .05, **p < .01, ***p < .001 and ****p < .0001.

The experiments interfere osteoblast differentiation with different degrees of CTS, to explore how mechanical stess regulates osteoblast differentiation through miR‐187‐3p/CNR2 pathway. These treatments included using a miR‐187‐3p mimic, CNR2 plasmid, cyclic tensile strain, or a combination of these factors. The application of cyclic tensile strain and the upregulation of CNR2 resulted in increased expression of Runx2 and OPN mRNA, which counteracted the inhibitory effects of miR‐187‐3p overexpression on osteogenic differentiation (Figure 5G–I). Additionally, osteoblasts overexpressing CNR2 and undergoing osteogenic differentiation showed elevated levels of Runx2 and OPN expression. Furthermore, we found that mechanical stress enhanced the osteogenic ability of osteoblasts by promoting the expression of CNR2 (Figure 5J). Moreover, ALP staining and quantitative analysis indicated that overexpressing CNR2 can inversed miR‐187‐3p mimic accelerated osteogenic differentiation. Furthermore, the impact of CTS intervention on osteogenic differentiation was even more pronounced. Respectively, in osteoblasts undergoing osteogenic differentiation (Figure 5K).

## 4 DISCUSSION

Osteoporosis is characterized by a disruption of bone homeostasis, a decrease in bone mass, and degeneration of bone microstructure.^41^, ^42^ The maintenance of bone health relies on the dynamic balance between bone formation and bone resorption. Disruption of this balance can lead to bone diseases.^43^ Postmenopausal women, who often experience reduced estrogen levels and increased osteoclast activity, are at risk of developing PMOP.^44^ Recent research suggests that moderate exercise can help prevent and manage PMOP.^45^, ^46^ Studies have shown that long‐term exercise can effectively prevent bone loss and enhance musculoskeletal health in postmenopausal female athletes.^47^ The essence of the exercise is to stimulate bone tissue through mechanical stress, promote the differentiation of osteoblast progenitor cells into osteoblasts, and inhibit the formation of osteoclasts. This ultimately leads to an increase in bone mass and the maintenance of bone homeostasis,^48^ significantly improving the bone health of patients with osteoporosis and enhancing their overall quality of life. While current clinical studies recommend various exercises for the prevention and treatment of osteoporosis, the exact mechanism underlying these benefits is still unclear.

To eliminate age‐related factors and simulate the mechanical stress of PMOP during exercise, this study utilized 8‐week‐old female C57BL/6J mice as an ovariectomy model. The results demonstrated that, at the conclusion of the experiment, the treadmill group exhibited significantly higher levels of BV/TV, Tb.N, and Tb.Th in cancellous bone compared to the control group. Additionally, the Tb.Sp of the treadmill group was significantly lower than that of the control group. These findings were further supported by HE and Masson staining, which revealed a higher number and volume of trabeculae in the treadmill group. Thus, running was found to increase Tb.BV/TV, Tb.N, and Tb.Th of trabecular bone, indicating that exercise promotes bone mass and can prevent osteoporosis in OVX mice. Moreover, our study involved the extraction and measurement the relative RNA and protein of bone tissue from the distal femur, and analysis the CNR2 expression in distal femoral bone tissue in immunohistochemistry, as well as immunofluorescence intensity. It was observed that treadmill exercise reduced the expression of miR‐187‐3p and increased the expression of CNR2 in OVX mice. This, in turn, enhanced bone volume and the expression of Runx2 and OPN osteogenic genes. These results suggest that exercise may have a potential role in preventing and treating postmenopausal osteoporosis by modulating the miR‐187‐3p/CNR2 pathway to promote bone formation in OVX mice, aligning closely with the OVX treadmill exercise model used in this study. The study outcomes lay a foundational groundwork for the clinical implementation of exercise for postmenopausal women.

In previous studies, miRNA plays a critical role in the regulation of bone metabolism, suggesting that abnormal expression of miRNA associated with bone metabolism may be closely linked to the development of bone‐related diseases like osteoporosis.^49^, ^50^ Studies have demonstrated that the expression of miR‐187‐3p is reduced during osteoblast activation mediated by titanium.^51^ Interestingly, an increase in miR‐187‐3p expression was observed in bone samples from non‐fracture patients with osteoporosis.^14^ These findings indicate the potential impact of miR‐187‐3p on bone formation. In our study, we have demonstrated that miR‐187‐3p inhibits the osteogenic differentiation of osteoblasts under mechanical stress. Furthermore, we have found that overexpressing miR‐187‐3p can counteract its inhibitory effect on osteoblast differentiation.

The process of bone remodeling is influenced by estrogen and endogenous cannabinoids. In postmenopausal individuals, the decline in ovarian function inhibits osteoblast differentiation, leading to decreased bone mass. This mechanism may be linked to decreased estrogen levels, resulting in reduced FAAH activity, lower endogenous cannabinoid levels, and decreased expression of osteogenic differentiation markers.^52^, ^53^ Recent research identified significant bone loss and cortical dilation in mice lacking the CB2 receptor.^28^ Activation of the CB2 receptor promotes osteogenic differentiation and mineralization of bone marrow mesenchymal stem cells,^40^ emphasizing the importance of CNR2 signaling in maintaining normal bone mass. The regulation of CNR2 signaling could potentially enhance osteogenic differentiation of bone stem cells. Notably, miR‐187‐3p was found to suppress CB2 receptor expression in hFOB 1.91 cells, inhibiting osteogenic differentiation. Furthermore, a double luciferase reporter gene assay confirmed that miR‐187‐3p directly targets CB2 receptor.^26^ These suggested that the CNR2 pathway may play a role in regulating the recruitment of osteocyte precursors to sites of bone formation.

The tensile stress of skeletal muscle contraction and compressive stress from weight‐bearing are key mechanical stimuli for bone. Due to study limitations, only the tensile stress on bone tissue cells during exercise was simulated. Numerous studies have shown that mechanical stress is a significant factor in promoting osteogenic differentiation.^54^ For instance, CTS regulates fibroblast proliferation and early osteogenic differentiation through integrin αVβ3.^55^ Mechanical stretching promotes osteogenesis of hJBMMSCs by inhibiting NF‐κB in a non‐inflammatory setting.^56^ It also induces an antioxidant response in BMSCs, reducing intracellular ROS and enhancing osteogenesis.^57^ Hence, we aimed to investigate the potential involvement of miR‐187‐3p and CNR2 in the early stages of osteogenic differentiation induced by cyclic tensile stress. Our findings demonstrate that mechanical stress enhances the expression of CNR2 and promotes osteogenic differentiation by suppressing the expression of miR‐187‐3p in the osteoblast nucleus. Consequently, we propose that miR‐187‐3p/CNR2 plays a significant role in the initial phases of mechanical stress‐induced osteogenic differentiation.

In this study, we applied CTS (10%, 0.5 Hz, 6 h/d) to osteoblasts in 3 and 7 days,^58^, ^59^ detected the mRNA and protein level Runx2, OPN, and ALP and alkaline phosphatase activity in osteoblasts and in response to CTS after 7 days, and verified the effects of CTS on osteoblasts phenotype (Figure 4A–E). All findings demonstrate that CTS is involved in the regulation of the osteoblasts phenotype by downregulating the expression of miR‐187‐3p. When the osteoblasts were stimulated by the CTS, the expression of miR‐187‐3p was decreased. Meanwhile, the expression of the CNR2 was increased. To find out whether miR‐187‐3p through CNR2 involved in the osteoblast osteogenic differentiation (Figure 4F–I), we overexpressed and knockdown it and detected the expression of the CNR2 and the osteogenic differentiation markers Runx2 and OPN, and found that all of them downregulated and upregulated. miR‐187‐3p was shown to inhibit mRNA and protein levels of Runx2, OPN, and CNR2 and alkaline phosphatase activity and mineralization in osteoblasts (Figure 5A–F). Besides, when transfected both miR‐187‐3p mimic and OV‐CNR2, the mRNA and protein expression of Runx2, OPN, and CNR2 and alkaline phosphatase activity were upregulated than that of single of overexpression miR‐187‐3p. When the cyclic tensile stress is loaded, there will be more significant upregulation results. (Figure 5G–K), which means miR‐187‐3p and CNR2 have directly interaction in this process. All of these results indicated that CNR2 is a target gene of mmiR‐187‐3p, and miR‐187‐3p target‐regulating CNR2 played a crucial role in the process of CTS promotes osteoblasts to differentiate.

Currently, there is a lack of research on the impact of mechanical stress and CNR2 regulation on miR‐187‐3p in osteoblasts. This study has provided initial evidence that miR‐187‐3p plays a role in enhancing osteoblast differentiation and is influenced by CTS. These findings suggest that miR‐187‐3p could be a crucial factor in the modulation of CNR2 expression in osteoblasts through CTS may have potential benefits in preventing and treating bone diseases, including postmenopausal osteoporosis. Further clinical studies are required to address this issue and explore potential solutions.

There are still some limitations that need to be addressed. The animal and cell experiments revealed that mechanical stress impacts large and complex molecules downstream of the CNR2 signal pathway, leading to macroscopic effects. Further research is required to target specific downstream signal molecules and proteins to understand their detailed roles. Moreover, due to the limitation of the experimental equipment FX‐5000, we can only simulate the tensile stress of osteoblasts in bone tissue during treadmill exercise, but lack the verification of compressive stress on osteoblast differentiation during treadmill exercise, highlighting the need for long‐term studies using different type of the mechanical stress models. For example, investigating whether compressive stress affects osteoblasts and delays OP development in mice with ovariectomy.

## 5 CONCLUSION

This study investigated the impact of mechanical stress on bone mass maintenance and osteoblast differentiation. The findings suggest that mechanical stress promotes osteoblast differentiation through the regulation of the miR‐187‐3p/CNR2 signaling pathway. Furthermore, future research could explore the potential of mechanical stress in the downstream pathway of miR‐187‐3p/CNR2 for the prevention and treatment of postmenopausal osteoporosis.

## AUTHOR CONTRIBUTIONS

Lei Zhang and Canjun Zeng contributed equally to this work and shared first authorship. Lei Zhang, Canjun Zeng, Juanyu Huang, and Haohang Yan were involved in conducting the experiments, analyzed the results, and contributed to manuscript writing. Lei Zhang, Canjun Zeng, and Runguang Li conducted the cyclic strain stress experiments to elucidate the miR‐187‐3p/CNR2 pathway in modulating osteoblast differentiation. Lei Zhang and Runguang Li conducted in vitro experiments to determine the functional mechanism of mechanical stress in treating postmenopausal osteoporosis. Lei Zhang, Canjun Zeng, Juanyu Huang, Haohang Yan, Yutao Jiang, and Runguang Li conceived the idea for the project. Lei Zhang, Canjun Zeng, and Runguang Li assisted with revising the figures and manuscript.

## FUNDING INFORMATION

This work was funded by the Natural Science Foundation of Guangdong Province, Grant/Award Number: 2019A1515012176 and 2022A1515012479, and The President Foundation of The Third Affiliated Hospital, Southern Medical University, Grant/Award Number: YM202209 and YP202216.

## DISCLOSURES

The authors declare that there are no conflicts of interest.

## Supporting information


Table S1.

## Data Availability

The data supporting the findings of this study are available from the corresponding author, RunGuang Li, upon reasonable request.

## References

[1] Gao L , Li S , Yue H , Zhang Z . Associations of serum cathepsin K and polymorphisms in CTSK gene with bone mineral density and bone metabolism markers in postmenopausal Chinese women. Front Endocrinol (Lausanne). 2020;11:48. 10.3389/fendo.2020.00048 PMC7031211 32117071

[2] Park E , Lee CG , Lim E , et al. Osteoprotective effects of loganic acid on osteoblastic and osteoclastic cells and osteoporosis‐induced mice. Int J Mol Sci. 2020;22(1):233. 10.3390/ijms22010233 PMC7795511 33379387

[3] Maredziak M , Smieszek A , Chrzastek K , Basinska K , Marycz K . Physical activity increases the total number of bone‐marrow‐derived mesenchymal stem cells, enhances their osteogenic potential, and inhibits their adipogenic properties. Stem Cells Int. 2015;2015:379093. 10.1155/2015/379093 PMC4488015 26167185

[4] Tong X , Chen X , Zhang S , et al. The effect of exercise on the prevention of osteoporosis and bone angiogenesis. Biomed Res Int. 2019;2019:8171897. 10.1155/2019/8171897 PMC6500645 31139653

[5] Wang L , Li JY , Zhang XZ , et al. Involvement of p38MAPK/NF‐kappaB signaling pathways in osteoblasts differentiation in response to mechanical stretch. Ann Biomed Eng. 2012;40(9):1884‐1894. 10.1007/s10439-012-0548-x 22441665

[6] Rath B , Nam J , Knobloch TJ , Lannutti JJ , Agarwal S . Compressive forces induce osteogenic gene expression in calvarial osteoblasts. J Biomech. 2008;41(5):1095‐1103. 10.1016/j.jbiomech.2007.11.024 PMC2291547 18191137

[7] Zeng Z , Yin X , Zhang X , Jing D , Feng X . Cyclic stretch enhances bone morphogenetic protein‐2‐induced osteoblastic differentiation through the inhibition of Hey1. Int J Mol Med. 2015;36(5):1273‐1281. 10.3892/ijmm.2015.2354 PMC4601743 26647760

[8] Mai Z , Peng Z , Wu S , et al. Single bout short duration fluid shear stress induces osteogenic differentiation of MC3T3‐E1 cells via integrin beta1 and BMP2 signaling cross‐talk. PLoS One. 2013;8(4):e61600. 10.1371/journal.pone.0061600 PMC3623893 23593489

[9] Thompson WR , Rubin CT , Rubin J . Mechanical regulation of signaling pathways in bone. Gene. 2012;503(2):179‐193. 10.1016/j.gene.2012.04.076 PMC3371109 22575727

[10] Liu P , Tu J , Wang W , et al. Effects of mechanical stress stimulation on function and expression mechanism of osteoblasts. Front Bioeng Biotechnol. 2022;10:830722. 10.3389/fbioe.2022.830722 PMC8893233 35252138

[11] Xu N , Cui G , Zhao S , et al. Therapeutic effects of mechanical stress‐induced C2C12‐derived exosomes on glucocorticoid‐induced osteoporosis through miR‐92a‐3p/PTEN/AKT signaling pathway. Int J Nanomedicine. 2023;18:7583‐7603. 10.2147/IJN.S435301 PMC10725637 38106447

[12] Bartel DP . MicroRNAs: genomics, biogenesis, mechanism, and function. Cell. 2004;116(2):281‐297. 10.1016/s0092-8674(04)00045-5 14744438

[13] Qiu Y , Zhu G , Zeng C , et al. Next‐generation sequencing of miRNAs and lncRNAs from rat femur and tibia under mechanical stress. Mol Med Rep. 2021;24(2): 561. 10.3892/mmr.2021.12200 PMC8201655 34109424

[14] Garmilla‐Ezquerra P , Sanudo C , Delgado‐Calle J , et al. Analysis of the bone microRNome in osteoporotic fractures. Calcif Tissue Int. 2015;96(1):30‐37. 10.1007/s00223-014-9935-7 25432767

[15] Mulrane L , Madden SF , Brennan DJ , et al. miR‐187 is an independent prognostic factor in breast cancer and confers increased invasive potential in vitro. Clin Cancer Res. 2012;18(24):6702‐6713. 10.1158/1078-0432.CCR-12-1420 23060431

[16] Yan ZX , Wu LL , Xue K , et al. MicroRNA187 overexpression is related to tumor progression and determines sensitivity to bortezomib in peripheral T‐cell lymphoma. Leukemia. 2014;28(4):880‐887. 10.1038/leu.2013.291 24104394

[17] Fei D , Zhao K , Yuan H , Xing J , Zhao D . MicroRNA‐187 exerts tumor‐suppressing functions in osteosarcoma by targeting ZEB2. Am J Cancer Res. 2016;6(12):2859‐2868. PMC5199759 28042505

[18] Dou C , Liu Z , Xu M , et al. miR‐187‐3p inhibits the metastasis and epithelial‐mesenchymal transition of hepatocellular carcinoma by targeting S100A4. Cancer Lett. 2016;381(2):380‐390. 10.1016/j.canlet.2016.08.011 27544906

[19] Alsharafi WA , Xiao B , Abuhamed MM , Bi FF , Luo ZH . Correlation between IL‐10 and microRNA‐187 expression in epileptic rat hippocampus and patients with temporal lobe epilepsy. Front Cell Neurosci. 2015;9:466. 10.3389/fncel.2015.00466 PMC4667084 26696826

[20] Locke JM , Da SXG , Dawe HR , Rutter GA , Harries LW . Increased expression of miR‐187 in human islets from individuals with type 2 diabetes is associated with reduced glucose‐stimulated insulin secretion. Diabetologia. 2014;57(1):122‐128. 10.1007/s00125-013-3089-4 PMC3855472 24149837

[21] Taylor HJ , Hung YH , Narisu N , et al. Human pancreatic islet microRNAs implicated in diabetes and related traits by large‐scale genetic analysis. Proc Natl Acad Sci USA. 2023;120(7):e2206797120. 10.1073/pnas.2206797120 PMC9963967 36757889

[22] Ren Z , Xie P , Lv J , et al. miR‐187‐3p inhibitor attenuates cerebral ischemia/reperfusion injury by regulating Seipin‐mediated autophagic flux. Int J Mol Med. 2020;46(3):1051‐1062. 10.3892/ijmm.2020.4642 PMC7387098 32705147

[23] Li XQ , Yu Q , Zhang ZL , Sun XJ , Ma H . MiR‐187‐3p mimic alleviates ischemia‐reperfusion‐induced pain hypersensitivity through inhibiting spinal P2X7R and subsequent mature IL‐1beta release in mice. Brain Behav Immun. 2019;79:91‐101. 10.1016/j.bbi.2019.05.021 31100367

[24] Yang D , Wei GY , Li M , et al. Cyclic tensile strain facilitates proliferation and migration of human aortic smooth muscle cells and reduces their apoptosis via miRNA‐187‐3p. Bioengineered. 2021;12(2):11439‐11450. 10.1080/21655979.2021.2009321 PMC8810176 34895047

[25] Xu Y , Sun L , Hu J , Xu S . Knockdown of hsa‐circ‐0001275 reverses dexamethasone‐induced osteoblast growth inhibition via mediation of miR‐377/CDKN1B axis. PLoS One. 2021;16(5):e0252126. 10.1371/journal.pone.0252126 PMC8158950 34043680

[26] Xu A , Yang Y , Shao Y , Wu M , Sun Y . Inhibiting effect of microRNA‐187‐3p on osteogenic differentiation of osteoblast precursor cells by suppressing cannabinoid receptor type 2. Differentiation. 2019;109:9‐15. 10.1016/j.diff.2019.07.002 31352121

[27] Fan L , Yang K , Yu R , Hui H , Wu W . circ‐Iqsec1 induces bone marrow‐derived mesenchymal stem cell (BMSC) osteogenic differentiation through the miR‐187‐3p/Satb2 signaling pathway. Arthritis Res Ther. 2022;24(1):273. 10.1186/s13075-022-02964-x PMC9749292 36517907

[28] Ofek O , Karsak M , Leclerc N , et al. Peripheral cannabinoid receptor, CB2, regulates bone mass. Proc Natl Acad Sci USA. 2006;103(3):696‐701. 10.1073/pnas.0504187103 PMC1334629 16407142

[29] Sophocleous A , Marino S , Kabir D , Ralston SH , Idris AI . Combined deficiency of the Cnr1 and Cnr2 receptors protects against age‐related bone loss by osteoclast inhibition. Aging Cell. 2017;16(5):1051‐1061. 10.1111/acel.12638 PMC5595693 28752643

[30] Clouse G , Penman S , Hadjiargyrou M , Komatsu DE , Thanos PK . Examining the role of cannabinoids on osteoporosis: a review. Arch Osteoporos. 2022;17(1):146. 10.1007/s11657-022-01190-x 36401719

[31] Schurman LD , Lu D , Kendall DA , Howlett AC , Lichtman AH . Molecular mechanism and cannabinoid pharmacology. Handb Exp Pharmacol. 2020;258:323‐353. 10.1007/164_2019_298 PMC8637936 32236882

[32] Tudorancea IM , Ciorpac M , Stanciu GD , et al. The therapeutic potential of the endocannabinoid system in age‐related diseases. Biomedicine. 2022;10(10): 2492. 10.3390/biomedicines10102492 PMC9599275 36289755

[33] Liu H , Gu R , Huang Q , et al. Isoliensinine suppresses osteoclast formation through NF‐kappaB signaling pathways and relieves ovariectomy‐induced bone loss. Front Pharmacol. 2022;13:870553. 10.3389/fphar.2022.870553 PMC9353689 35935862

[34] Zhao Y , Zou B , Shi Z , Wu Q , Chen GQ . The effect of 3‐hydroxybutyrate on the in vitro differentiation of murine osteoblast MC3T3‐E1 and in vivo bone formation in ovariectomized rats. Biomaterials. 2007;28(20):3063‐3073. 10.1016/j.biomaterials.2007.03.003 17418401

[35] Li L , Chen X , Lv S , et al. Influence of exercise on bone remodeling‐related hormones and cytokines in ovariectomized rats: a model of postmenopausal osteoporosis. PLoS One. 2014;9(11):e112845. 10.1371/journal.pone.0112845 PMC4231162 25393283

[36] Fang C , Qiao Y , Mun SH , et al. Cutting edge: EZH2 promotes osteoclastogenesis by epigenetic silencing of the negative regulator IRF8. J Immunol. 2016;196(11):4452‐4456. 10.4049/jimmunol.1501466 PMC4875854 27183582

[37] Di Benedetto A , Sun L , Zambonin CG , et al. Osteoblast regulation via ligand‐activated nuclear trafficking of the oxytocin receptor. Proc Natl Acad Sci USA. 2014;111(46):16502‐16507. 10.1073/pnas.1419349111 PMC4246276 25378700

[38] Chen Z , Zhao F , Liang C , et al. Silencing of miR‐138‐5p sensitizes bone anabolic action to mechanical stimuli. Theranostics. 2020;10(26):12263‐12278. 10.7150/thno.53009 PMC7667683 33204341

[39] Khan AU , Qu R , Yang Y , et al. Lamin A/C‐dependent translocation of megakaryoblastic leukemia‐1 and beta‐catenin in cyclic strain‐induced osteogenesis. Cells. 2021;10(12): 3518. 10.3390/cells10123518 PMC8700688 34944031

[40] Sun YX , Xu AH , Yang Y , Zhang JX , Yu AW . Activation of cannabinoid receptor 2 enhances osteogenic differentiation of bone marrow derived mesenchymal stem cells. Biomed Res Int. 2015;2015:874982. 10.1155/2015/874982 PMC4317596 25685815

[41] Moon YJ , Zhang Z , Bang IH , et al. Sirtuin 6 in preosteoclasts suppresses age‐ and estrogen deficiency‐related bone loss by stabilizing estrogen receptor alpha. Cell Death Differ. 2019;26(11):2358‐2370. 10.1038/s41418-019-0306-9 PMC6889517 30787391

[42] Xu D , Liu J , Zheng W , Gao Q , Gao Y , Leng X . Identification of polysaccharides from *Dipsacus asperoides* and their effects on osteoblast proliferation and differentiation in a high‐glucose environment. Front Pharmacol. 2022;13:851956. 10.3389/fphar.2022.851956 PMC8986998 35401194

[43] Yu H , Zhang X , Song W , et al. Effects of 3‐dimensional bioprinting alginate/gelatin hydrogel scaffold extract on proliferation and differentiation of human dental pulp stem cells. J Endod. 2019;45(6):706‐715. 10.1016/j.joen.2019.03.004 31056297

[44] Sozen T , Ozisik L , Basaran NC . An overview and management of osteoporosis. Eur J Rheumatol. 2017;4(1):46‐56. 10.5152/eurjrheum.2016.048 PMC5335887 28293453

[45] Mohan S , Bhat CG , Wergedal JE , Kesavan C . In vivo evidence of IGF‐I‐estrogen crosstalk in mediating the cortical bone response to mechanical strain. Bone Res. 2014;2:14007. 10.1038/boneres.2014.7 PMC4472140 26273520

[46] Kawao N , Kawaguchi M , Ohira T , et al. Renal failure suppresses muscle irisin expression, and irisin blunts cortical bone loss in mice. J Cachexia Sarcopenia Muscle. 2022;13(1):758‐771. 10.1002/jcsm.12892 PMC8818650 34997830

[47] Moreira LD , Oliveira ML , Lirani‐Galvao AP , et al. Physical exercise and osteoporosis: effects of different types of exercises on bone and physical function of postmenopausal women. Arq Bras Endocrinol Metabol. 2014;58(5):514‐522. 10.1590/0004-2730000003374 25166042

[48] Ma D , Wu L , He Z . Effects of walking on the preservation of bone mineral density in perimenopausal and postmenopausal women: a systematic review and meta‐analysis. Menopause. 2013;20(11):1216‐1226. 10.1097/GME.0000000000000100 24149921

[49] Yang Y , Yujiao W , Fang W , et al. The roles of miRNA, lncRNA and circRNA in the development of osteoporosis. Biol Res. 2020;53(1):40. 10.1186/s40659-020-00309-z PMC7493179 32938500

[50] De Martinis M , Ginaldi L , Allegra A , et al. The osteoporosis/microbiota linkage: the role of miRNA. Int J Mol Sci. 2020;21(23): 8887. 10.3390/ijms21238887 PMC7727697 33255179

[51] Palmieri A , Pezzetti F , Avantaggiato A , et al. Titanium acts on osteoblast translational process. J Oral Implantol. 2008;34(4):190‐195. 10.1563/0.869.1 18780563

[52] Dong A , He K , Dudok B , et al. A fluorescent sensor for spatiotemporally resolved imaging of endocannabinoid dynamics in vivo. Nat Biotechnol. 2022;40(5):787‐798. 10.1038/s41587-021-01074-4 PMC9091059 34764491

[53] El HM , Ninh VK , El HE , Bradley JM , Gardner JD . Estrogen receptor antagonism exacerbates cardiac structural and functional remodeling in female rats. Am J Physiol Heart Circ Physiol. 2017;312(1):H98‐H105. 10.1152/ajpheart.00348.2016 PMC5283916 27769996

[54] Kang KS , Lee SJ , Lee HS , Moon W , Cho DW . Effects of combined mechanical stimulation on the proliferation and differentiation of pre‐osteoblasts. Exp Mol Med. 2011;43(6):367‐373. 10.3858/emm.2011.43.6.040 PMC3128915 21532314

[55] Peng Y , Qu R , Feng Y , et al. Regulation of the integrin alphaVbeta3‐ actin filaments axis in early osteogenesis of human fibroblasts under cyclic tensile stress. Stem Cell Res Ther. 2021;12(1):523. 10.1186/s13287-021-02597-y PMC8496073 34620239

[56] Chen X , Liu Y , Ding W , et al. Mechanical stretch‐induced osteogenic differentiation of human jaw bone marrow mesenchymal stem cells (hJBMMSCs) via inhibition of the NF‐kappaB pathway. Cell Death Dis. 2018;9(2):207. 10.1038/s41419-018-0279-5 PMC5833399 29434225

[57] Chen X , Yan J , He F , et al. Mechanical stretch induces antioxidant responses and osteogenic differentiation in human mesenchymal stem cells through activation of the AMPK‐SIRT1 signaling pathway. Free Radic Biol Med. 2018;126:187‐201. 10.1016/j.freeradbiomed.2018.08.001 PMC6165675 30096433

[58] Zhang R , Wan J , Wang H . Mechanical strain triggers differentiation of dental mesenchymal stem cells by activating osteogenesis‐specific biomarkers expression. Am J Transl Res. 2019;11(1):233‐244. PMC6357315 30787982

[59] Yang X , Cai X , Wang J , et al. Mechanical stretch inhibits adipogenesis and stimulates osteogenesis of adipose stem cells. Cell Prolif. 2012;45(2):158‐166. 10.1111/j.1365-2184.2011.00802.x PMC6496762 22229452

