# Supplementary material for: Exploration of the miR‐187‐3p/CNR2 pathway in modulating osteoblast differentiation and treating postmenopausal osteoporosis through mechanical stress
Source: FASEB J. 2024 Jul 3;38(13):e23776. doi: 10.1096/fj.202400113RR (PMC13688052; doi:10.1096/fj.202400113RR)
Supplement: Supplementary file 1 [file FSB2-38-e23776-s001.docx]

**Table 1 Primer sequence**

| **Target Gene** | **Primer Sequence** |
| --- | --- |
| miR-187-3p | F:TCGTGTCTTGTGTTGCAGCC |
|  | R:GTGCAG GGTCCGAGGT |
| ALP | F:CGTCTCCATGGTGGATTATGCT |
|  | R:TGGATGTCCTTTACCGTCGTG |
| Runx2 | F:TGGCTTGGGTTTCAGGTTAG |
|  | R:GGTTTCTTAGGGTCTTGGAGTG |
| OPN | F:TCCCTCGATGTCATCCCTGT |
|  | R:ATCACATCCGACTGATCGGC |
| CNR2 | F:AGCCCTCATACCTGTTCATTGG |
|  | R:GTGAAGGTCATAGTCACGCTG |
| GADPH | F:AGGTCGG TGTGAACGGATTTG |
|  | R:TGTAGACCATGTAGTTGAGGTCA |
| U6 | F:CGCTTCGGCAGCACATATACTAAAATTGGAAC |
|  | R:GCTTCACGAATTTG CGTGTCATCCTTGC |
